# Lupanine Improves Glucose Homeostasis by Influencing K_ATP_ Channels and Insulin Gene Expression

**DOI:** 10.3390/molecules201019085

**Published:** 2015-10-20

**Authors:** Mats Wiedemann, Carmen M. Gurrola-Díaz, Belinda Vargas-Guerrero, Michael Wink, Pedro M. García-López, Martina Düfer

**Affiliations:** 1 Department of Pharmaceutical and Medicinal Chemistry, Münster University, Corrensstraße 48, 48149 Münster, Germany; E-Mail: matswiedemann@uni-muenster.de; 2 Departamento de Biología Molecular y Genómica, Centro Universitario de Ciencias de la Salud, Universidad de Guadalajara, 44340 Guadalajara, Jalisco, Mexico; E-Mails: carmenhpv@yahoo.de (C.M.G.-D.); bevargro@hotmail.com (B.V.-G.); 3 Institute of Pharmacy and Molecular Biotechnology, Heidelberg University, Heidelberg 69120, Germany; E-Mail: Wink@uni-heidelberg.de; 4 Departamento de Botánica y Zoología, Centro Universitario de Ciencias Biológicas y Agropecuarias, Universidad de Guadalajara, 45110 Guadalajara, Jalisco, Mexico; E-Mail: pgarcia@cucba.udg.mx

**Keywords:** quinolizidine alkaloids, beta-cell, insulin secretion, K_ATP_ channel, lupanine, *Lupinus*, membrane potential, glucose tolerance, streptozotocin, type-2 diabetes mellitus

## Abstract

The glucose-lowering effects of lupin seeds involve the combined action of several components. The present study investigates the influence of one of the main quinolizidine alkaloids, lupanine, on pancreatic beta cells and in an animal model of type-2 diabetes mellitus. *In vitro* studies were performed with insulin-secreting INS-1E cells or islets of C57BL/6 mice. In the *in vivo* experiments, hyperglycemia was induced in rats by injecting streptozotocin (65 mg/kg body weight). In the presence of 15 mmol/L glucose, insulin secretion was significantly elevated by 0.5 mmol/L lupanine, whereas the alkaloid did not stimulate insulin release with lower glucose concentrations. In islets treated with l-arginine, the potentiating effect of lupanine already occurred at 8 mmol/L glucose. Lupanine increased the expression of the *Ins-1* gene. The potentiating effect on secretion was correlated to membrane depolarization and an increase in the frequency of Ca^2+^ action potentials. Determination of the current through ATP-dependent K^+^ channels (K_ATP_ channels) revealed that lupanine directly inhibited the channel. The effect was dose-dependent but, even with a high lupanine concentration of 1 mmol/L or after a prolonged exposure time (12 h), the K_ATP_ channel block was incomplete. Oral administration of lupanine did not induce hypoglycemia. By contrast, lupanine improved glycemic control in response to an oral glucose tolerance test in streptozotocin-diabetic rats. In summary, lupanine acts as a positive modulator of insulin release obviously without a risk for hypoglycemic episodes.

## 1. Introduction

The dramatically rising number of patients suffering from type-2 diabetes mellitus (T2DM) is one of the most urgent problems challenging health systems all over the world. In order to enlarge the portfolio of glucose-lowering drugs, research investigating the anti-diabetic potential of plants or plant extracts is continuously gaining weight. Especially the developing countries of South America or Africa that are disadvantaged by having only limited access to evidence-based, expensive treatment of metabolic syndrome or T2DM might benefit from natural products with anti-diabetic properties.

Human and animal studies provide evidence that extracts of some *Lupinus* species or lupin seeds decrease plasma glucose concentration in patients with glucose intolerance, manifest T2DM or in animal models with experimentally induced diabetes [1,2,3]. Antihyperglycemic effects are caused by the lupin seed protein γ-conglutin as well as by several quinolizidine alkaloids [4,5]. Fornasini *et al.* [3] demonstrated in a small study that the effect of *Lupinus mutabilis* (*L. mutabilis*) increases with increasing dysglycemia. The data revealed a reduction in plasma glucose and insulin concentrations ~90 min after application of the drug in a capsuled formulation in patients with a fasting glucose concentration above 100 mg/dL. A phase II clinical trial published by the same group [2] including patients with recently diagnosed T2DM demonstrated the efficacy of both, cooked *L. mutabilis* or the alkaloid-containing extract. Importantly, the study was performed with formulations that contained a very low concentration of alkaloids (2.5 mg/kg body weight) to avoid any side effects based on the toxicity of quinolizidine alkaloids. Lupin alkaloids are mainly neurotoxins that affect nicotinic and muscarinic acetylcholine receptors and Na^+^ and K^+^ channels [6,7,8].

Intravenous injection of sparteine increased plasma insulin concentration in subjects with T2DM without affecting insulin sensitivity [9]. Experiments with isolated rat islets confirmed a direct stimulatory effect on insulin release for sparteine, lupanine and its 13-α-hydroxy- or 17-oxo-derivative as well as for the synthetic derivative 2-thionosparteine [10,11]. Key steps for glucose-induced insulin secretion are the generation of reduction equivalents during glycolysis, mitochondrial ATP synthesis inducing closure of ATP-dependent K^+^ channels (K_ATP_ channels) and subsequent opening of voltage-dependent Ca^2+^ channels (Ca_v_ channels) triggering exocytosis of insulin-containing granules. In addition, there are several ways to augment insulin release, e.g., by mechanisms elevating intracellular cAMP concentration [12]. As the stimulatory effect of the above-mentioned alkaloids is partly reversed by the K_ATP_ channel opener diazoxide [11] interference with the K_ATP_ channel-dependent pathway seems to be involved.

The present study investigates the mode of action of lupanine on electrical activity of pancreatic beta cells, insulin secretion and insulin gene expression. Furthermore, the influence of lupanine on glucose tolerance and insulin sensitivity was tested in an *in vivo* model for type-2 diabetes mellitus.

## 2. Results and Discussion

### 2.1. Lupanine Lowers Plasma Glucose Concentration and Improves Glycemic Control in an in Vivo Model for Diabetes

**Figure 1:**
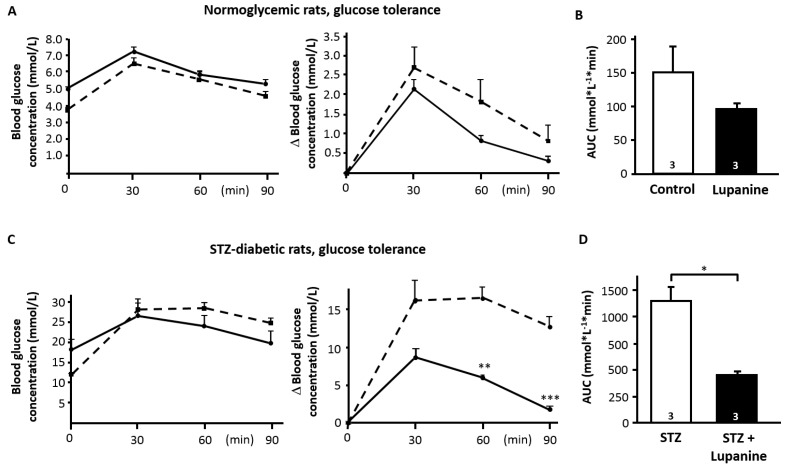

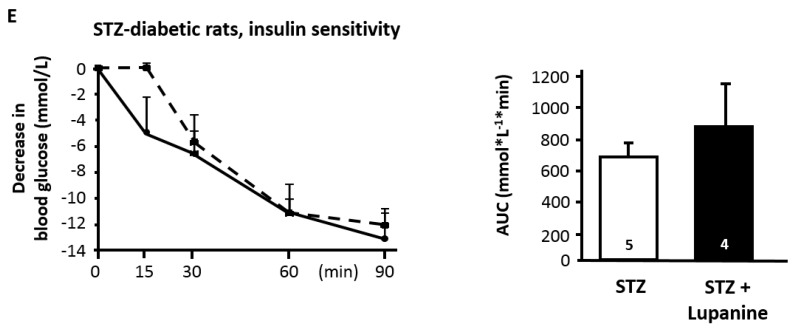
Influence of lupanine on glycemic control. Changes in blood glucose concentration in response to an oral application of 2 g glucose/kg BW (**A**–**D**) or in response to intraperitoneal injection of 0.5 I.U. insulin/kg BW (**E**); (**A**) Diagram showing the changes in blood glucose concentration (left panel: absolute values, right panel: changes from baseline). The dashed line illustrates the data during the oGTT of control rats, the continuous line represents the results for lupanine-treated rats (20 mg/kg BW, oral administration 30 min before oGTT); (**B**) Calculation of the area under the curve (AUC) for a period of 90 min after application of the glucose bolus for control (white bar) and lupanine-treated (black bar) animals; (**C**,**D**) Same maneuver as described for (**A**,**B**) but performed with STZ-diabetic rats. STZ-treatment drastically impaired glucose tolerance (compare white bars in (**B**,**D**)). Change of blood glucose concentration (**C**) and AUC (**D**) were significantly improved by the administration of lupanine; (**E**) Insulin-induced reduction of blood glucose concentration in STZ-diabetic rats without (dashed line) or with lupanine (continuous line) treatment. The number of animals is given in the bars of (**B**,**D**,**E**). * *p* ≤ 0.05, ** *p* ≤ 0.01, *** *p* ≤ 0.001.

To characterize the acute effect of lupanine, 20 mg/kg body weight (BW) of the alkaloid were orally administered in non-diabetic and diabetic rats. After 30 min, an oral glucose tolerance test (oGTT, glucose: 2 g/kg BW) was performed. This dosage of lupanine did not lower blood glucose, which was 4.4 ± 0.2 mg/dL before and 4.9 ± 0.2 mg/dL 30 min after administration of lupanine (*n* = 3). In comparison to control animals, the rise in plasma glucose concentration during the oGTT expressed as area under the curve (AUC) tended to be reduced in lupanine-treated rats but the effect was not significant (Figure 1A,B). To test whether lupanine effectively improves glucose tolerance in diabetic animals, the acute, low-dose streptozotocin (STZ) protocol was used. This series of experiments revealed a beneficial effect of lupanine in diabetic animals (Figure 1C). Glucose tolerance was improved 60 and 90 min after administration of the glucose bolus. In agreement with a better glycemic control, the AUC was significantly smaller in lupanine-treated rats compared to untreated diabetic animals (Figure 1D). Determination of insulin sensitivity revealed that lupanine did not change the response of STZ-diabetic rats to exogenous insulin (0.5 I.U./kg BW, intraperitoneal injection) in comparison to untreated control animals (Figure 1E).

### 2.2. Lupanine Influences the Expression of Ins-1 Gene

**Figure 2:**
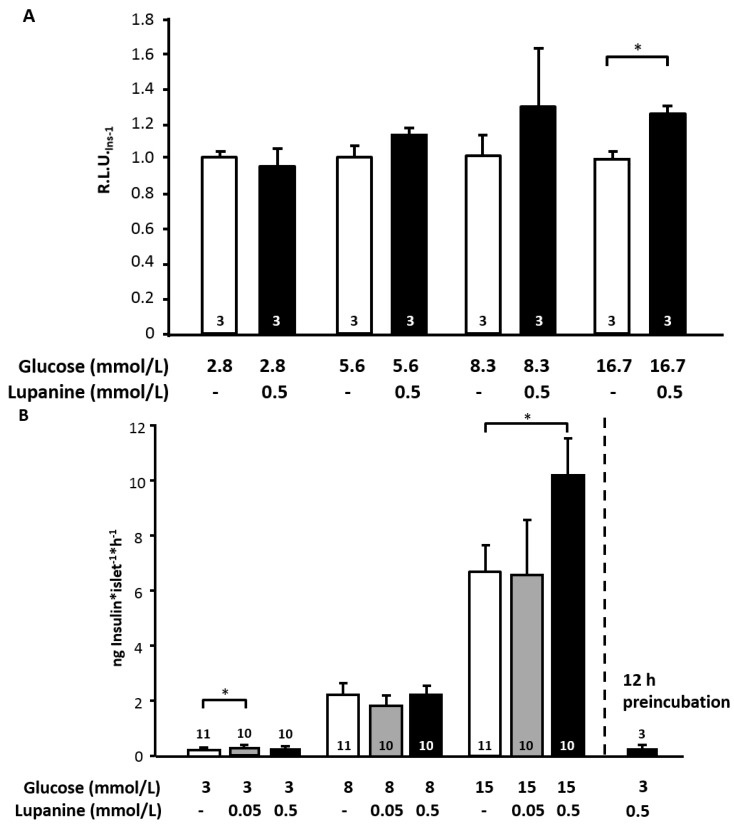
Lupanine affects the expression of *Ins-1* mRNA and insulin secretion. (**A**) INS-1E cells were incubated with lupanine (0.5 mmol/L) for 30 min in the presence of the indicated glucose concentrations; (**B**) Murine islets were incubated with 0.05 or 0.5 mmol/L lupanine for 60 min in the presence of the indicated glucose concentrations (left bars). The bar on the right depicts basal insulin release after 12 h preincubation with 0.5 mmol/L lupanine. The number of independent experiments is given in the bars. * *p* ≤ 0.05.

To test whether lupanine affects the expression of insulin the insulin-secreting clonal rat-derived cell line INS-1E was used. Incubation of INS-1E cells with 0.5 mmol/L lupanine for 30 min showed that lupanine was ineffective at glucose concentrations up to 8.3 mmol/L. On the contrary, in the presence of 16.7 mmol/L glucose, a significant rise in the *Ins-1* mRNA level by ~25% was observed (Figure 2A).

### 2.3. Lupanine Increases Glucose-Induced Insulin Release

Islets were stimulated with 8 and 15 mmol/L glucose in the presence or absence of lupanine. Lupanine (0.05 and 0.5 mmol/L) did not affect insulin secretion in islets treated with 8 mmol/L glucose. By contrast, insulin secretion induced by 15 mmol/L glucose was potentiated by 0.5 mmol/L lupanine to ~140% (Figure 2B). The influence of lupanine on basal insulin release was also investigated. Under substimulatory conditions (3 mmol/L glucose), we observed a slight rise in insulin release by the low concentration of 0.05 mmol/L, whereas 0.5 mmol/L were without any effect when applied acutely and after a prolonged exposure time of 12 h. A further increase to 1 mmol/L lupanine was accompanied by a small decrease of basal insulin secretion (0.38 ± 0.11 *vs.* 0.25 ± 0.07 ng/(islet·h) insulin, *n* = 5, *p* ≤ 0.05).

### 2.4. Lupanine Reduces the Current through K_ATP_ Channels and Modifies Electrical Activity of Beta Cells

**Figure 3:**
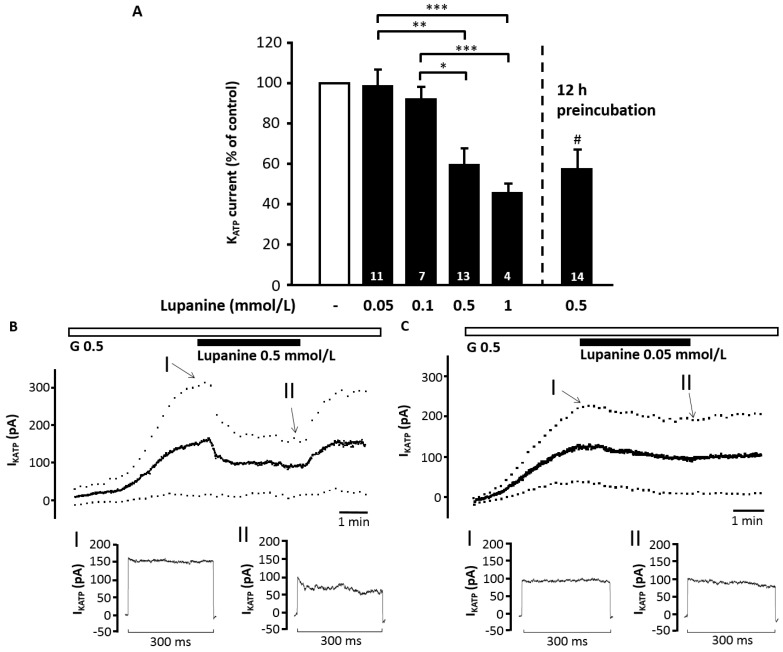
Lupanine reduces the K_ATP_ current. K_ATP_ current of isolated islet cells was determined in the standard whole-cell configuration, *i.e.*, without cell metabolism. Bath solution contained 0.5 mmol/L glucose (G 0.5). (**A**) Summary of the dose–response relation for the acute application of 0.05–1 mmol/L lupanine (left bars) or the effect of preincubation with 0.5 mmol/L lupanine for 12 h (right bar). Values are normalized to the current recorded under control conditions directly before changing bath solution and in the preincubation experiments to the current of untreated control cells. The two experiments illustrate representative recordings with 0.5 (**B**) and 0.05 (**C**) mmol/L lupanine. The time points marked with “I, II” are shown below the traces in higher temporal resolution. The number of cells tested for each condition is given in the bars of the diagram. * *p* ≤ 0.05, ** *p* ≤ 0.01, *** *p* ≤ 0.001, ^#^ *p* ≤ 0.05 *vs.* untreated control cells.

To check whether the stimulatory action of the alkaloid is mediated by an effect on the stimulus-secretion cascade measurements of ion currents and plasma membrane potential (V_m_) were performed. Lupanine had no effect on K_ATP_ current at a concentration range of 0.05 to 0.1 mmol/L but dose-dependently and reversibly inhibited the current at concentrations of 0.5 mmol/L (from 123 ± 14 pA to 73 ± 12 pA, *n* = 13, *p* ≤ 0.001) and 1 mmol/L (from 176 ± 42 pA to 84 ± 27 pA, *n* = 4, *p* ≤ 0.01) (Figure 3A–C). In cells incubated with 0.5 mmol/L lupanine for 12 h the current was reduced to a similar extent as obtained by acute treatment (control cells: 119 ± 34 pA, *n* = 12 *vs.* lupanine-pretreated cells: 70 ± 14 pA, *n* = 14, *p* ≤ 0.05). As these experiments were performed in the standard whole-cell configuration (*i.e.*, without cell metabolism), the data indicate a direct interaction of lupanine with K_ATP_ channels. The K_ATP_ channel opener diazoxide (0.1 mmol/L) only transiently antagonized the inhibitory effect of 1 mmol/L lupanine (K_ATP_ current in the presence of lupanine: 84 ± 27 pA, maximal increase after addition of diazoxide: 110 ± 29 pA, *n* = 4, *p* ≤ 0.01). Control experiments with tolbutamide confirmed that the current determined by this protocol was exclusively K_ATP_ current (control: 177 ± 23 pA, after addition of 100 µM tolbutamide: 4 ± 0.4 pA, *n* = 6, *p* ≤ 0.01).

**Figure 4:**
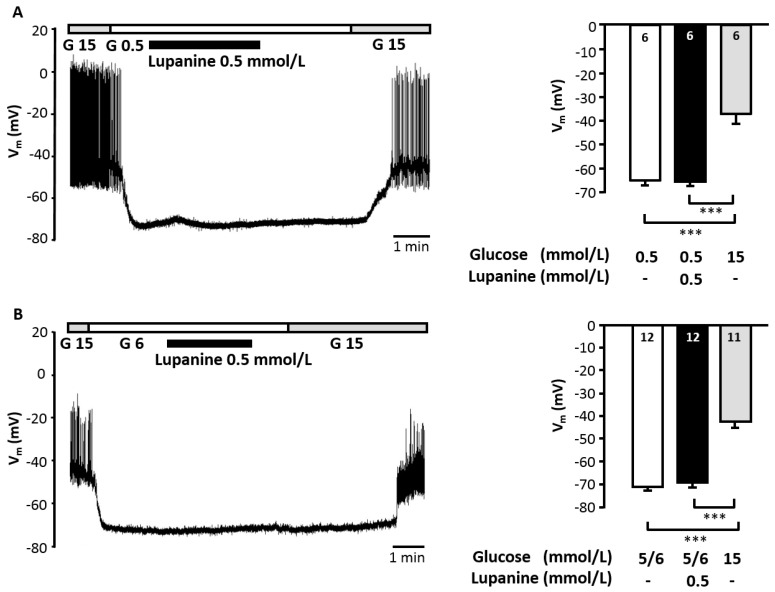
Lupanine has no effect on V_m_ of unstimulated beta cells. Cells were stimulated with 15 mmol/L glucose (G 15) at the beginning and the end of each experiments to verify metabolic integrity. Lupanine (0.5 mmol/L) was added in the presence of 0.5 mmol/L glucose (**A**) or at a glucose concentration of 5–6 mmol/L (**B**), which is close to the threshold for induction of electrical activity. Under both conditions lupanine did not depolarize the cells. The left part of (**A**,**B**) shows representative recordings with 0.5 mmol/L (G 0.5) and 6 mmol/L (G 6) glucose, respectively. The right part summarizes the data. White bars: V_m_ in the presence of 0.5 or 5–6 mmol/L glucose, black bars: + lupanine, grey bars: depolarization of V_m_ induced by 15 mmol/L glucose at the end of the experiments. The number of cells tested for each condition is given in the bars of the diagram. *** *p* ≤ 0.001.

Based on the results obtained for insulin release the inhibitory effect of 0.5 mmol/L lupanine on K_ATP_ current should not be large enough to induce electrical activity *per se*. However, it might already affect V_m_. To test for this, V_m_ was measured in unstimulated beta cells with intact metabolism. Lupanine did not affect V_m_ in bath solution with low glucose (0.5 mmol/L) or at glucose concentrations close to the threshold for activation (5–6 mmol/L glucose) (Figure 4A,B). As 0.05 mmol/L lupanine slightly elevated insulin secretion in bath solution supplemented with 3 mmol/L glucose, V_m_ was also determined under these conditions. These experiments showed that the effect on basal insulin release was not mediated by any changes in V_m_ which was −69.8 ± 1.7 mV in the presence of 3 mmol/L glucose and −70.0 ± 1.8 mV after addition of 0.05 mmol/L lupanine (*n* = 12).

**Figure 5:**
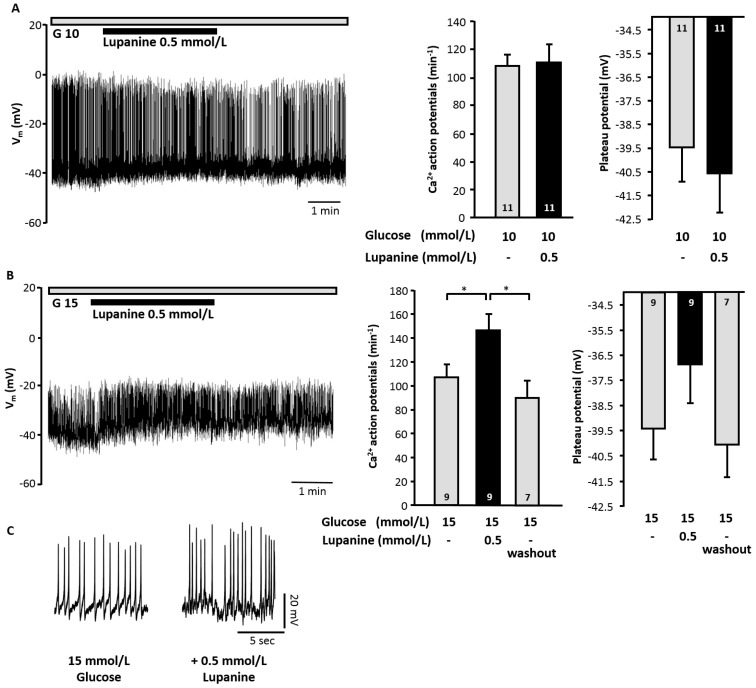
Lupanine increases electrical activity of glucose-stimulated beta cells. Lupanine has no effect on V_m_ and on the frequency of Ca^2+^ action potentials in the presence of 10 mmol/L glucose (G 10) (**A**) but increases the frequency of action potentials in the presence of 15 mmol/L glucose (G 15) (**B**). The left part of (**A**,**B**) shows representative recordings, in the middle of (**A**,**B**) analysis of action potential frequency is summarized in the diagrams. The diagrams on the right illustrate the influence of lupanine on the plateau potential, *i.e.*, the potential at which Ca^2+^ action potentials start; and (**C**) illustrates changes in action potentials of the series of experiments presented in (**B**) in a higher temporal resolution. The number of cells tested for each condition is given in the bars of the diagrams. * *p* ≤ 0.05.

To investigate whether the potentiating effect of lupanine on insulin secretion in glucose-stimulated islets was caused by alterations in V_m_ we tested the influence of lupanine on beta cells that were already electrically active. The frequency of Ca^2+^ action potentials was unaffected by 0.5 mmol/L lupanine in the presence of 10 mmol/L glucose (Figure 5A) but—in agreement with the results obtained for insulin release—increased by ~40% in the presence of 15 mmol/L glucose (Figure 5B,C). Similar to the inhibitory influence on K_ATP_ currents the effects of lupanine on action potential frequency was reversible (Figure 5B). Correspondingly, the plateau potential (potential at which Ca^2+^ action potentials start) was slightly more depolarized after addition of lupanine. This increase occurred in all experiments but was not statistically different.

### 2.5. Lupanine Potentiates the Influence of l-Arginine on Insulin Release

**Figure 6:**
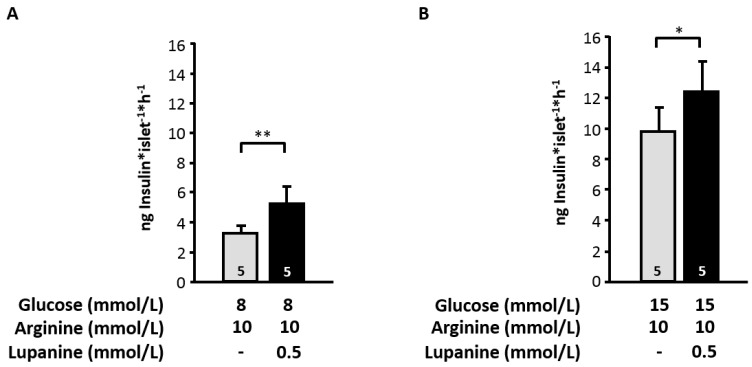
Effect of lupanine in combination with l-arginine on insulin secretion. Murine islets were incubated with 0.5 mmol/L lupanine for 60 min in the presence of 10 mmol/L l-arginine and 8 mmol/L glucose (**A**) or 15 mmol/L glucose (**B**). The number of independent experiments is given in the bars. * *p* ≤ 0.05, ** *p* ≤ 0.01.

As our data indicate that lupanine acts by augmentation of electrical activity we tested whether potentiation of insulin release is shifted to lower glucose concentrations when cells are already partly depolarized by l-arginine. Lupanine (0.5 mmol/L) in combination with l-arginine (10 mmol/L) had no effect in the presence of 3 mmol/L glucose (0.47 ± 0.12 *vs.* 0.39 ± 0.17 ng/(islet·h) insulin, *n* = 5) but elevated insulin release in the presence of 8 and 15 mmol/L glucose (Figure 6).

Our *in vivo* investigation shows that lupanine improves glucose homeostasis in STZ-diabetic animals but not in normoglycemic controls. These data are in agreement with human studies which also demonstrate that the effect of *Lupinus* raw material or extracts depends on the glycemic status of the patients [3]. The lack of effect on insulin sensitivity suggests that lupanine interacts with the endocrine pancreas. Analysis of beta cell stimulus-secretion coupling revealed that lupanine directly inhibits K_ATP_ channels. The transient, antagonizing effect of diazoxide most likely indicates competition for the same binding sites. The effect of lupanine is dose-dependent but importantly even with the high concentration of 1 mmol/L only ~50% of the current are blocked. This observation explains why lupanine does not depolarize V_m_ to the threshold for opening of Ca_v_ channels in the presence of low glucose concentrations. Obviously, the remaining K^+^ conductance is large enough to maintain V_m_ hyperpolarized. However, when beta cells are already electrically active, *i.e.*, when membrane resistance is high due to glucose- or arginine-mediated stimulation, further reduction of K_ATP_ current can potentiate membrane depolarization resulting in a rise in action potential frequency. With respect to our *in vivo* data and the studies with humans these characteristics may explain why the effect of lupanine increases with rising hyperglycemia. Another possibility is that lupanine might exert additional effects on the amplifying pathway. The fact that lupanine does not act as a primary stimulus is very important regarding its potential as an antidiabetic drug. It is well known that hypoglycemia induced by K_ATP_ channel inhibitors like sulfonylureas or glinides and the associated complications are limiting the clinical value of these drugs [13,14]. K_ATP_ channel inhibition has also been shown for the lupin alkaloid sparteine in the insulin-secreting cell line HIT-T15 and in murine beta cells [10,15]. In contrast to lupanine the inhibitory effect of sparteine was much more pronounced and large enough to induce insulin secretion at micromolar concentrations in unstimulated islets [10]. For skeletal muscle cells, it has been shown that lupanine inhibits voltage-dependent Na^+^ channels (Na_v_ channels) at concentrations similar to those of our study [7]. However, as in rodent beta cells, glucose-induced action potentials are solely carried by Ca^2+^ [16] we can exclude any contribution of interactions with Na_v_ channels. Our preincubation experiments show that after prolonged exposure to lupanine inhibition of K_ATP_ channels persists even in the absence of the alkaloid. This most likely indicates membrane enrichment of the compound. Importantly, the extent of inhibition is similar to the acute effect of lupanine and basal insulin secretion is not affected. These observations suggest that this degree of reduction in K_ATP_ current represents a saturated condition that is not cumulative during increasing exposure time.

Of note, lupanine is not only acting on the stimulus-secretion cascade but also affects the expression of insulin mRNA. The elevated gene transcription level may contribute to a better secretory response especially during long-term treatment with the drug. At present, we cannot explain the small but significant alterations in basal insulin release observed with 0.05 and 1 mmol/L lupanine, respectively. As lupanine does not influence V_m_ in the presence of 0.5–6 mmol/L glucose in a concentration range from 0.05 to 1 mmol/L these changes are not linked to a V_m_-dependent pathway and may result from direct interference with the exocytotic machinery. Additional effects not-related to interactions with ion channels have also been suggested to be involved in the action of sparteine on insulin release [10].

Lupins gain increasing significance as functional foods. When studying lupin components one must be aware of its limitations with respect to therapeutic use. The neurotoxic quinolizidine alkaloids are known for its hazardous potential inducing trembling, seizures and disturbance of blood pressure regulation [17,18,19]. Case reports also point to anticholinergic symptoms after ingestion of bitter lupin flour [20]. In the majority of studies adverse effects are most prominent for sparteine [18,21]. In a feeding study with *Lupinus angustifolius* [22], no toxic effects were observed in rats over a period of 90 days. The main alkaloids of this variety were identified as lupanine and 13-hydroxylupanine and the daily intake was calculated to range from 400 to 500 mg alkaloid/kg BW. For isolated lupanine toxicity studies revealed an LD_50_ of 174–177 mg/kg BW (intraperitoneal application) and 1664 mg/kg BW (oral intake) in mice and rats [19], *i.e.*, at a concentration ~80-fold higher compared to the 20 mg/kg BW used in our *in vivo* experiment. Human studies emphasizing the potential of lupin raw material for improvement of glycemic control do not point to drug-related adverse effects [2,3] but mostly sweet lupins with low alkaloid contents were used. However, as different *Lupinus* species vary substantially with respect to their alkaloid content and alkaloid composition [21,23], standardization is very important especially considering the use of lupin-based products to support control of glucose homeostasis.

To avoid unforeseeable effects pure lupanine or lupanine-enriched supplements might be more appropriate. Importantly, analysis of protein isolates obtained by different methods from different lupin varieties (*L. albus* and *L. angustifolius*) showed that these isolates all contained low amounts (<0.002%) of alkaloids [24]. As it is known that the protein γ-conglutin improves glucose transport [25] and elevates pancreatic insulin content [26] it is tempting to speculate whether combined application of lupanine and γ-conglutin act in synergy. Our *in vivo* data clearly show that lupanine improves glucose tolerance in response to glucose ingestion. Further studies are needed to investigate whether lupanine interacts with the incretine system. In agreement with the results obtained for stimulation of insulin release the effects of lupanine get important when blood glucose levels are pathologically high. Bobkiewicz-Kozlowska [4] reported that approximately the same concentration of lupanine as used in our present study did not lower blood glucose in non-diabetic or STZ-diabetic rats without any glucose challenge over a period of 2 h. This is in agreement with our data where blood glucose at the beginning of the oGTT (*i.e.*, 30 min after lupanine-injection) was not reduced and only a tendency to improved glucose tolerance was observed in the oGTT with non-diabetic animals.

## 3. Experimental Section

### 3.1. Cell and Islet Preparation, INS-1E Cell Culture

Experiments were performed with islets of Langerhans or dispersed islet cells from adult C57BL/6N mice (Charles River, Sulzfeld, Germany). The principles of laboratory animal care were followed according to German laws. Mice were euthanized with CO_2_, thereafter pancreatic tissue was digested by collagenase, islets were cultured as a whole or trypsinized to obtain single cells or small cell clusters. Culture was performed in RPMI 1640 medium (11.1 mmol/L glucose) supplemented with 10% fetal calf serum, 100 U/mL penicillin and 100 µg/mL streptomycin for up to 1 week.

The clonal rat-derived β cell line INS-1E, derived from parental INS-1 cells, was kindly donated by S. Ullrich (Tübingen University, Germany) previously authorized by C. Wollheim (University Medical Center, Switzerland). Cells were cultivated in 75 cm^2^ culture bottles with 20 mL of RPMI 1640 medium supplemented with 5% heat-inactivated fetal calf serum, 1 mmol/L sodium pyruvate, 50 μmol/L 2-mercaptoethanol, 2 mmol/L glutamine, 10 mmol/L HEPES, 100 U/mL penicillin, and 100 μg/mL streptomycin. Cells were passaged in sterile conditions by gentle trypsinization once a week. Fresh medium was added every five days.

All cultures were kept at 37 °C in 5% CO_2_ humidified atmosphere.

### 3.2. In Vivo Experiments

For glucose and insulin tolerance tests, male Wistar rats (200–250 g) were housed at 25 °C, 65%–70% of relative humidity under 12 h light-darkness cycles with *ad libitum* access to a standard rodent diet (Purina LabDiet^®^ 5001) and water. The local ethics committee approved this protocol and all animal procedures were conducted in accordance with the production, care, and use of laboratory animals established in the Mexican Official Standard.

For experimental induction of diabetes, animals received a single intraperitoneal injection of 65 mg/kg BW streptozotocin diluted in acetate buffer (100 mmol/L, pH 4.5) after 15 h of food deprivation. Forty-eight hours post-induction, glucose levels were measured to verify the diabetic stage (glycemia > 200 mg/dL). Diabetic animals were divided randomly into two groups (control *vs.* lupanine-treated).

The effect of lupanine was measured in fasted rats with and without experimental induction of diabetes. After 15 h of food deprivation and 30 min before the oGTT each animal was orally administered: lupanine, 20 mg/kg BW in 0.9% NaCl, or solely 0.9% NaCl as vehicle (control treatment). At time zero of the experiment, a glucose solution (2 g/kg BW) was orally administered to each rat by gavage through a metal cannula. Blood glucose was determined at 0, 30, 60, and 90 min after the glucose overload. For determination of insulin sensitivity STZ-treated rats were subjected to intraperitoneal insulin loading (0.5 I.U./kg BW) after a 12 h fasting period. Blood glucose concentration was measured at 15, 30, 60, and 90 min after insulin injection using a blood glucose meter (One Touch Ultra^®^, Johnson & Johnson, New Brunswick, NJ, USA). In the lupanine-group, lupanine (20 mg/kg BW) was given directly before administration of insulin (t_0_). Data were evaluated as changes from t_0_.

### 3.3. Electrophysiology

Patch-clamp experiments were performed with pipettes pulled from borosilicate glass capillaries (resistance of 3–5 MΩ when filled with pipette solution). Bath solution for recordings of K_ATP_ current or membrane potential (V_m_) was composed of (mmol/L): 140 NaCl, 5 KCl, 1.2 MgCl_2_, 2.5 CaCl_2_, glucose as indicated, and 10 mM HEPES, pH 7.4, adjusted with NaOH. Pipette solution for determination of whole-cell K_ATP_ current contained (mmol/L): 130 KCl, 4 MgCl_2_, 2 CaCl_2_, 10 EGTA, 0.65 Na_2_ATP and 20 mM HEPES, pH adjusted to 7.4 with KOH. For recordings in the perforated-patch configuration pipettes were filled with (mmol/L): 10 KCl, 10 NaCl, 70 mM K_2_SO_4_, 4 MgCl_2_, 2 CaCl_2_, 10 EGTA, 10 HEPES, and amphotericin B (250 μg/mL), pH 7.15, adjusted with KOH.

For data acquisition and analysis, an EPC-10 patch-clamp amplifier (HEKA, Lambrecht, Germany) and the software “Patchmaster” and “Fitmaster” were used. V_m_ was determined in the current clamp mode. K_ATP_ current was recorded by application of 300 ms pulses to −80 and −60 mV, respectively, starting from a holding potential of −70 mV at 15 s intervals [27]. The current elicited by this protocol was completely blocked by 100 µmol/L tolbutamide.

### 3.4. Insulin Gene Expression

INS-1E cells were seeded in 6-well tissue culture plates at a cell density of 8 × 10^5^ cells/well. Cells were maintained for 4–5 days before lupanine treatment. For the lupanine treatment, cells were pre-incubated with glucose-free-culture medium for 2 h. Then, cells were washed twice and incubated for 30 min at 37 °C with glucose-free-Krebs-Ringer-bicarbonate-HEPES-buffer (KRBH). Subsequently, cells were incubated for 30 min in KRBH and stimuli (2.8, 5.6, 8.3 and 16.7 mmo/L glucose with or without 0.5 mmol/L lupanine). Afterwards, plates were put on ice. RNA was isolated from harvested cells with the RNeasy^®^ Protect Mini Kit (Qiagen, Valencia, CA, USA). RNA (2 μg) was reverse-transcribed into cDNA using the Transcriptor First Strand cDNA Synthesis Kit (Roche, Mannheim, Germany), according to manufacturer’s instructions. *Ins-1* gene expression was determined by real-time PCR, as described elsewhere [26] by using a LightCycler^®^ FastStart DNA MasterPLUS SYBR Green I Kit (Roche, Germany). The *Rps 18* gene was used as an internal control. The threshold (Ct) values obtained for *Ins-1* gene were normalized against *Rps 18* Ct values. Relative quantification of PCR products was determined with the 2−^ΔΔCt^ method. A melting curve analysis was performed to verify that a single amplicon was amplified for each analyzed gene.

### 3.5. Insulin Secretion

After isolation islets were cultured for 24 h. Medium was removed and batches of 5 islets were incubated for 60 min at 37 °C in Krebs-Ringer-HEPES buffer, pH 7.4 adjusted with NaOH with different concentrations of glucose and/or lupanine as indicated. Insulin was determined in the supernatant by radioimmunoassay with rat insulin (Millipore, Billerica, MA, USA) as the standard.

### 3.6. Drugs and Chemicals

Lupanine (98% purity, determined by GC-MS spectra) was isolated from *Lupinus albus* in the Wink laboratory as described by Wink [28,29]. Collagenase P was from Roche Diagnostics (Mannheim, Germany), streptozotocin from Sigma-Aldrich (St. Louis, MI, USA), RPMI 1640 medium was from Life Technologies (Darmstadt, Germany), chemicals for buffer solutions were from Sigma-Aldrich (Taufkirchen, Germany or St Louis, MI, USA) or Diagonal (Münster, Germany).

### 3.7. Statistical Evaluation

Electrophysiological experiments are illustrated by representative recordings. For evaluation of K_ATP_ current the amplitude induced by stepping from −70 to −60 mV was analyzed. For determination of action potential frequency Ca^2+^ action potentials were counted and averaged for 30 s directly before changing the bath solution. For each series of experiments data of at least 3 independent preparations were used. Data are given as mean ± SEM. Statistical significance of differences was assessed by Student’s *t*-test for paired or unpaired (data presented in Figure 1, Figure 3 and Figure 6) values. For multiple comparisons analysis of variance (ANOVA) followed by Student-Newman-Keuls test was performed (data presented in Figure 2B, Figure 4 and Figure 5). *Ins-1* gene expression was expressed in relative light units (R.L.U.) and differences among groups were assessed with the Wilcoxon–Mann–Whitney Test (PASW statistical software version 18, Chicago, IL, USA, Figure 2A). *p* ≤ 0.05 was considered as significantly different.

## 4. Conclusions

Our study shows that lupanine potentiates glucose-stimulated insulin release by directly affecting K_ATP_ channels. In addition, the alkaloid increases insulin gene expression. The improvement of glucose tolerance in rats with experimentally induced hyperglycemia underlines the antidiabetic potential of lupanine, which may be useful for supportive treatment of T2DM.
